# *KIR2DL5* mutation and loss underlies sporadic dermal neurofibroma pathogenesis and growth

**DOI:** 10.18632/oncotarget.17736

**Published:** 2017-05-10

**Authors:** Corina Anastasaki, Sonika Dahiya, David H. Gutmann

**Affiliations:** ^1^ Department of Neurology, Washington University School of Medicine, St. Louis, MO 63110, USA; ^2^ Department of Pathology, Washington University School of Medicine, St. Louis, MO 63110, USA

**Keywords:** sporadic neurofibroma, Schwann cells, KIR2DL5, tumor suppressor gene, RAS/AKT/mTOR signaling

## Abstract

Dermal neurofibromas (DNFs) are benign peripheral nerve sheath tumors thought to originate from Schwann cell progenitors. These tumors represent one of the hallmarks of the neurofibromatosis type 1 (NF1) tumor predisposition syndrome, where they can number in the thousands. While NF1-DNFs arise due to mutations in the *NF1* gene, the vast majority of DNFs occur sporadically (sp-DNFs), where the genetic etiology is currently unknown. Herein, we employed whole-exome sequencing of sp-DNFs to identify a recurrent mutation in the *KIR2DL5* gene, which codes for a protein suppressor of natural killer (NK) cell activity. While the function of KIR2DL5 outside of the immune system is unknown, we identified a *KIR2DL5*^N173D^ mutation in three of nine sp-DNFs, resulting in loss of KIR2DL5 protein expression. In contrast, two of these subjects had unrelated tumors, which retained KIR2DL5 protein expression. Moreover, loss of KIR2DL5 expression was demonstrated in 15 of 45 independently-identified sp-DNFs. Consistent with its potential role as a negative growth regulator important for neurofibroma maintenance, ectopic *KIR2DL5*^N173D^ expression in normal human Schwann cells resulted in reduced KIR2DL5 expression and increased cell proliferation. Similarly, *KIR2DL5* short hairpin RNA knockdown (KD) decreased KIR2DL5 protein levels and increased cell proliferation, as well as correlated with PDGFRβ and downstream RAS/AKT/mTOR hyperactivation. Importantly, inhibition of PDGFRβ or AKT/mTOR activity in KIR2DL5-KD human Schwann cells reduced proliferation to control levels. Collectively, these findings establish KIR2DL5 as a new Schwann cell growth regulator relevant to sp-DNF pathogenesis, which links sporadic and NF1-associated DNFs through RAS pathway hyperactivation.

## INTRODUCTION

Dermal neurofibromas (DNFs) are common low-grade peripheral nerve sheath tumors [1], which are hypothesized to arise from Schwann cell progenitors [2–4]. While these tumors do not undergo malignant transformation, they can be painful or number in the thousands, as seen in some adults with the neurofibromatosis type 1 (NF1; OMIM: 613113) cancer predisposition syndrome. Nearly all adults with NF1 manifest DNFs, which result from bi-allelic inactivation of the *NF1* tumor suppressor gene and loss of *NF1* protein (neurofibromin) expression [5–7]. This bi-allelic *NF1* loss reflects a combination of a germline *NF1* gene mutation and the acquisition of a somatic *NF1* mutation in Schwann cell lineage populations [3, 4]. As a RAS-GTPase-activating protein (RAS-GAP), neurofibromin loss in Schwann cells leads to increased RAS activation [8–10] and downstream mechanistic target of rapamycin (mTOR)-mediated hyperproliferation [11–14].

While *NF1* mutations characterize NF1-DNFs, the majority of DNFs occurs in the absence of NF1. These sporadic DNFs (sp-DNFs) are histologically identical to their NF1-associated counterparts, and most often arise as solitary dermal tumors. Since these tumors are encountered in individuals lacking other features supportive of a clinical diagnosis of NF1, they do not harbor *NF1* gene mutations. As such, it is currently not known what genetic mutations are responsible for the genesis of sp-DNFs. In an effort to identify potential molecular etiologies for these non-syndromal benign nerve sheath tumors, we performed whole exome sequencing on a series of NF1-associated and sporadic dermal neurofibromas.

## RESULTS AND DISCUSSION

**Figure 1:**
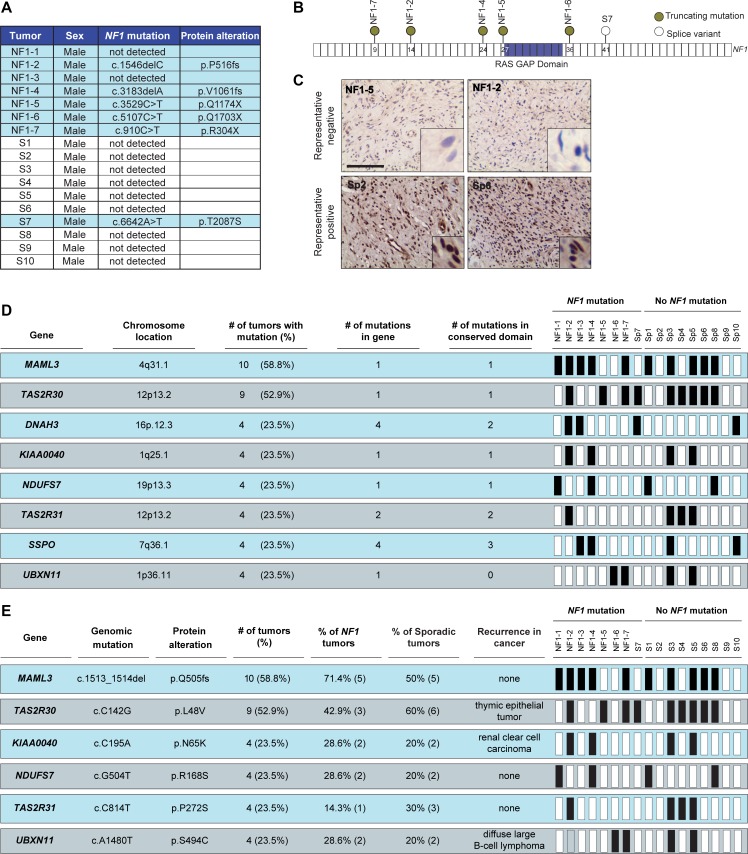
*NF1* gene mutation and neurofibromin expression stratify DNFs into two subgroups (**A**) List of detected *NF1* gene mutations and predicted protein alterations. (**B**) Schematic representation of *NF1* gene mutations. (**C**) Representative images depicting decreased neurofibromin expression in tumors with *NF1* mutations compared to sp-DNFs. Scale bar, 25 μm. (**D**) Genes recurrently mutated in more than four DNFs. (**E**) Recurrent mutations identified in more than four DNFs. Black boxes = mutation.

In order to identify potential molecular etiologies for sporadic DNFs (sp-DNFs), we examined a cohort of 17 male patients (7 NF1-DNFs and 10 sp-DNFs) by whole-exome sequencing. Using this approach, *NF1* gene mutations were detected in 5/7 NF1-DNFs, and surprisingly, 1/10 (Sp7) sp-DNFs where the identified point mutation is predicted to result in a splicing variant of the *NF1* transcript (Figure 1A, 1B). The inability to detect mutations in two of the NF1-DNFs likely reflects the insensitivity of exome sequencing to identify intronic mutations or large-gene deletions, both common mutational types in NF1 [15, 16]. However, consistent with a clinical diagnosis of NF1 or the presence of a *NF1* gene mutation, immunohistochemical staining confirmed loss of neurofibromin expression in the Sp7 tumor and in all NF1-DNFs (Figure 1C, Supplementary Figure 1). Subject Sp7 was lost to follow-up, and may have undiagnosed NF1 or harbored a tumor that underwent bi-allelic *NF1* gene inactivation [17]. Conversely, all sp-DNFs without an identified *NF1* mutation, as well as an independent cohort of 45 unrelated sp-DNFs retain neurofibromin expression (Supplementary Figure 1), consistent with exclusion of NF1 as a clinical diagnosis.

While 105 genes with exonic nucleotide variations (frameshift insertions/deletions, missense, and nonsense mutations; Supplementary Table 1) were identified in more than one DNF, eight genes were mutated in at least four DNFs (> 22.22% of the cohort; Figure 1D, Supplementary Figure 2). These mutated genes did not separate sporadic or NF1-associated DNFs into clear subgroups; however, the specific nucleotide alterations identified in 6 of the 8 genes were identical in all of the tumors examined (Figure 1E). Importantly, three of these mutations have also been reported in other cancers (cBioPortal; www.cbioportal.org), suggesting potential importance in DNF pathogenesis that could be pursued beyond the scope of this study.

**Figure 2:**
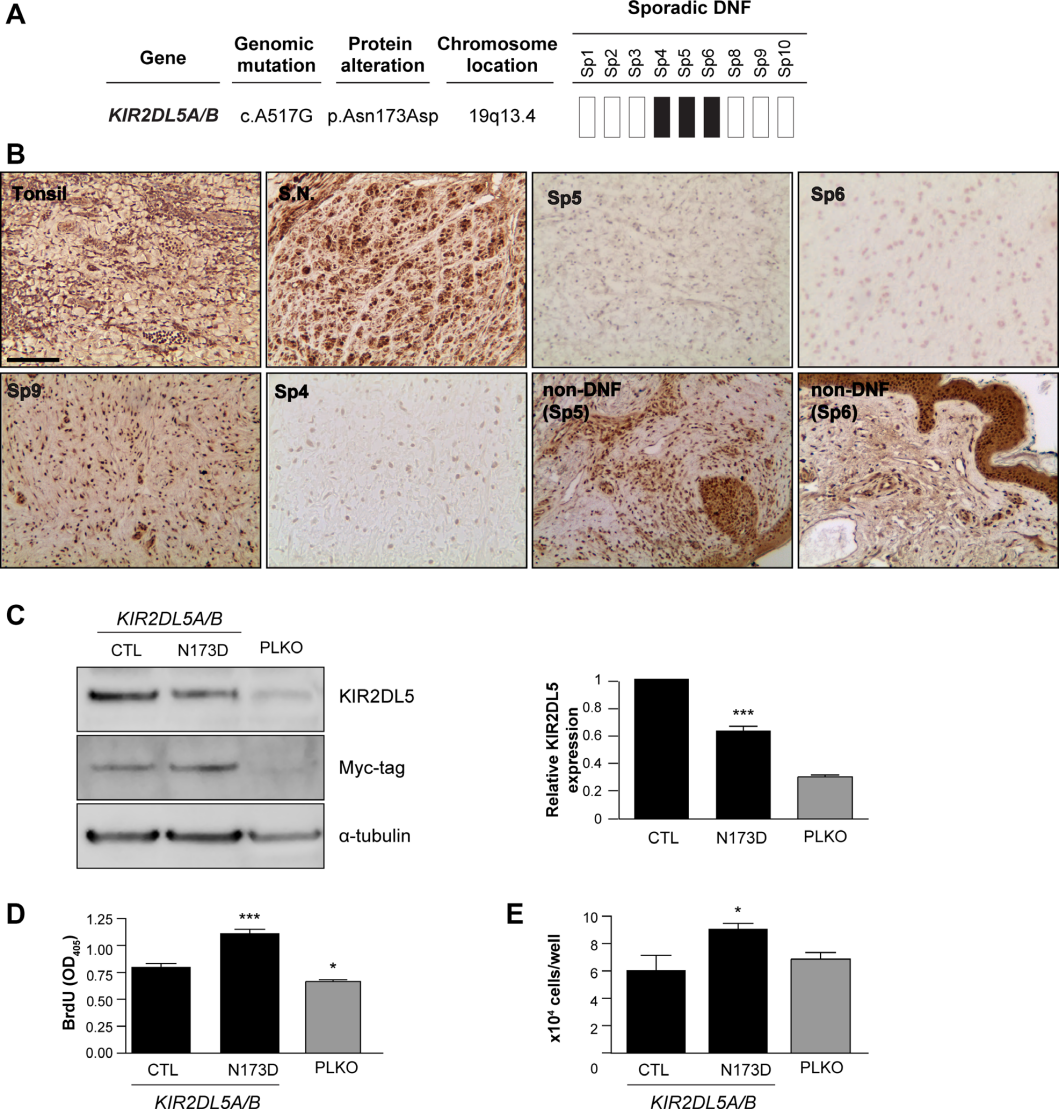
*KIR2DL5*^N173D^ mutation reduces KIR2DL5 expression (**A**) 3/9 sp-DNFs harbor the *KIR2DL5*^N173D^ mutation. (**B**) *KIR2DL5* mutation is associated with decreased KIR2DL5 protein levels in sp-DNFs but not NF1-DNFs or non-DNF tissue. Tonsil and sural nerve (S.N.) were used as controls. (**C**) *KIR2DL5*^N173D^ expression results in reduced KIR2DL5 levels, as demonstrated with a myc antibody immunoblot of human Schwann cells infected with a myc-tagged *KIR2DL5* ^WT^ *or a KIR2DL5* ^N173D^ construct. *KIR2DL5*^N173D^ expression results in increased (**D**) cell proliferation and (**E**) cell number relative to *KIR2DL5*^WT^ control-infected Schwann cells. PLKO-GFP-control (PLKO)-infected cells are shown for comparison. Data are represented as means of three independent infections/over-expression construct ± s.e.m. One-way ANOVA was used for statistical analysis. *p < 0.05; ***p < 0.001. Scale bar 50 μm.

While no mutations were found in genes commonly mutated in a related peripheral nerve sheath tumor (schwannoma; *NF2* [18, 19], *LZTR1* [20, 21], *SMARCB1* [22, 23] genes), recurrent mutations in the *KIR2DL*5 gene were identified *only* in the sp-DNFs (three of nine sp-DNFs examined). *KIR2DL5* is a gene unique to primates, and is not found in cattle or mice [24]. Of note, the particular *KIR2DL*5 mutation (Asn173Asp, N173D; 3/9 tumors) resulted in a non-conservative change in an evolutionarily-conserved residue (Figure 2A) within a predicted immunoglobulin-like domain [25] (Supplementary Figure 3A). In the three KIR2DL5^N173D^-mutant sp-DNFs, immunohistochemistry revealed no appreciable KIR2DL5 protein expression, whereas human tonsil, normal human sural nerve, and all NF1-DNFs examined expressed KIR2DL5 (Figure 2B). To determine whether loss of KIR2DL5 protein expression correlated with the presence of a KIR2DL5^N173D^ mutation, we examined two unrelated tumors (Sp5: basal cell carcinoma, Sp6: keratinous cyst) isolated from two of the three subjects with this mutation in their DNFs (Figure 2B). Both non-DNF tissues were immunopositive for KIR2DL5, suggesting that *KIR2DL5*^N173D^ is likely a DNF-specific mutation. Moreover, in an independent cohort of sp-DNFs, 15/45 tumors were KIR2DL5-immunonegative (33%; Figure 2B, Supplementary Figure 3B), further supporting the hypothesis that KIR2DL5 expression loss is specific to sp-DNFs and likely is caused by the *KIR2DL5*^N173D^ mutation.

To determine whether the *KIR2DL5*^N173D^ mutation was responsible for the reduction in KIR2DL5 protein expression observed, normal human Schwann cells were infected with either *KIR2DL5*^WT^ or *KIR2DL5*^N173D^ viral expression constructs (Figure 2D). Whereas wild-type KIR2DL5 expression was easily detected by Western blot and promoted a minor increase in Schwann cell growth (1.18-fold increase compared to PLKO-infected Schwann cells), *KIR2DL5*^N173D^mutant-expressing cells exhibited a 36.9% decrease in KIR2DL5 expression, a 1.88-fold increase in cell proliferation (BrdU incorporation) (Figure 2E), and a 1.5-fold increase in total cell numbers (Figure 2F) relative to *KIR2DL5*^WT^ expressing cells. Together, these data demonstrate that the *KIR2DL5*^N173D^ mutation functions in a dominant negative manner to regulate KIR2DL5 expression and Schwann cell growth.

KIRs were initially discovered on the surface of natural killer (NK) cells [26], where they have been reported to mediate the immune response of NK cells and a subset of T lymphocytes against infection and cancer [27–29]. While KIR2DL5 is an orphan receptor with no known ligands, its phosphorylation in NK cells recruits the Src homology region 2-containing protein tyrosine phosphatase-2 (SHP-2) molecule, resulting in activation of downstream signaling pathways. Since the role of KIR2DL5 has not been previously explored in non-immune system cells, we first demonstrated that KIR2DL5 is expressed in S100β^+^ Schwann cells within normal human peripheral (sural) nerve by immunofluorescence (Figure 2C), and in normal human Schwann cells by western blot (CTL; Figure 2D) and immunofluorescence (Supplementary Figure 4A). While the majority of the KIR2DL5 expression in both normal sural nerve and immunopositive sDNFs was found in the Schwann cell compartment, there was also expression in other cell types. In this regard, co-labelling of KIR2DL5^+^ cells with mast cell (tryptase, c-Kit) and fibroblast (vimentin)-specific antibodies (Supplementary Figure 4) revealed that the few remaining KIR2DL5-immunoreactive cells in the Sp4, Sp5 and Sp6 DNF tumor specimens were stromal elements.

**Figure 3:**
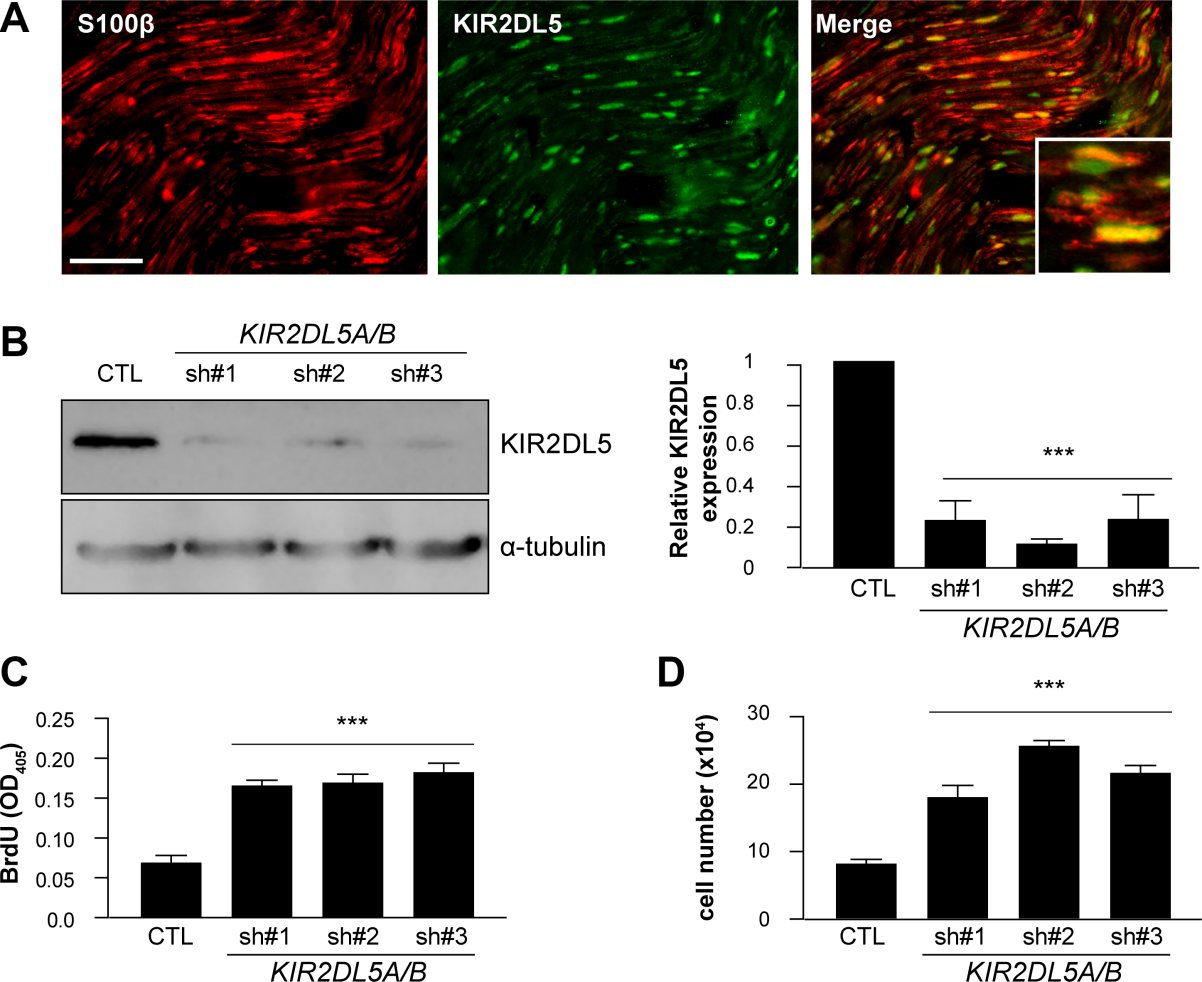
KIR2DL5 is a negative Schwann cell growth regulator (**A**) Co-localization of KIR2DL5 with S100β^+^ Schwann cells in normal human sural nerve. b. shRNA silencing of *KIR2DL5A/B* expression in normal human Schwann cells, where *KIR2DL5A/BKD* cell KIR2DL5 expression levels were calculated by normalizing the relative KIR2DL5/α-tubulin band intensity ratio to that of GFP-control-infected Schwann cells. (**B)** western blot). *KIR2DL5A/BKD* results in increased (**C**) cell number and (**D**) proliferation relative to GFP-infected serum-starved Schwann cells. Data are represented as means of three independent infections/shRNA construct ± s.e.m. One-way ANOVA with Bonferroni correction was used for statistical analysis. ***p < 0.001. Scale bar 25 μm.

As a complementary approach to expressing the mutant KIR2DL5 gene in normal human Schwann cells to reduce KIR2DL5 expression, endogenous *KIR2DL5* expression was also decreased by RNA (shRNA) interference. Since KIR2DL5 is encoded by two genes, *KIR2DL5A* (OMIM: 605305) and *KIR2DL5B* (OMIM: 615727) that are > 99% sequence identical [25], three independently-generated short-hairpin RNA molecules were employed to target both genes. Following Schwann cell infection, 76.78% (sh#1; TCTCTCCATGACTCACCCTAT), 88.29% (sh#2; CAGGAGCTCATTTGACATGTA) and 76.32% (sh#3; GAAACTCTTCAAGTAGTTCAT) *KIR2DL5* knockdown was achieved (Figure 3A), leading to 2.2, 3.2- and 2.7-fold increases in cell number, respectively (direct cell counting; Figure 3B) relative to GFP-vector-infected cells (CTL). This increase in cell numbers represented a 2.7- (sh#1), 2.83- (sh#2), and 3- (sh#3) fold increase in proliferation (BrdU incorporation; Figure 3C), with no change in apoptosis (TUNEL staining; Supplementary Figure 3C). Expression of control *KIR2DL5* engineered so as not to be targeted by these shRNA constructs following shRNA knockdown restored KIR2DL5 expression, as well as cell proliferation and cell numbers (Supplementary Figure 3D–3F).

**Figure 4:**
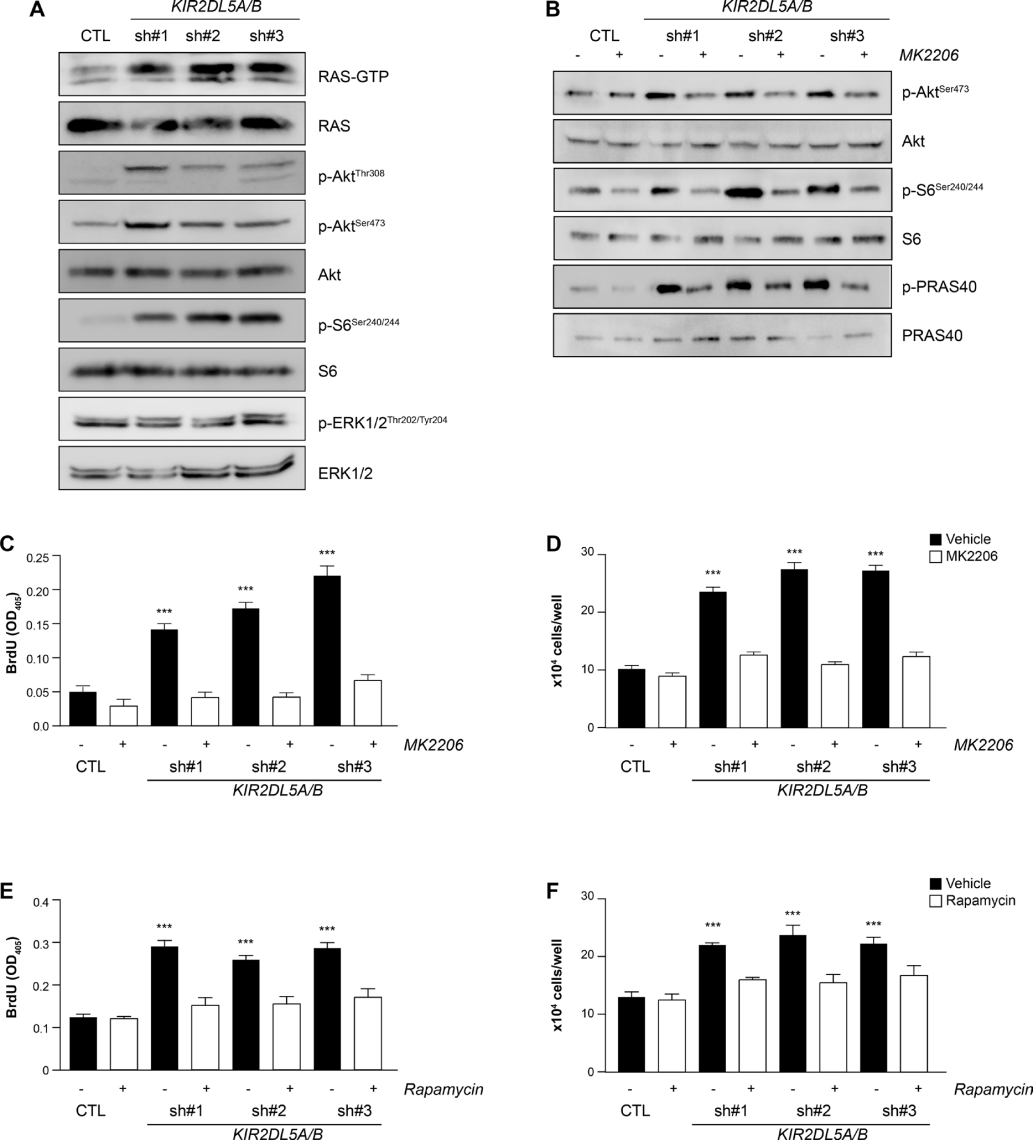
KIR2DL5A/B is a negative regulator of RAS/AKT signaling (**A**) shRNA silencing of *KIR2DL5A/B* in human Schwann cells results in increased RAS activity (RAS-GTP), as well as increased AKT^Thr308^, AKT^Ser473^, and S6^Ser240/244^, but not ERK1/2^Thr202/Tyr204^, phosphorylation relative to GFP-infected Schwann cells. (**B**) AKT^Ser473^, S6^Ser240/244^ and PRAS40^Thr246^ phosphorylation is restored to wild-type levels following MK2206 treatment of *KIR2DL5A/B*^KD^ Schwann cells. 4 h MK2206 (**C**–**D**) or rapamycin (**E**–**F**) treatment restores (C, E) cell number and (D, F) proliferation of *KIR2DL5A/B*^KD^ Schwann cells relative to GFP-infected controls. Experiments were repeated at least three times using independently-derived cell lysates. Data are represented as means ± s.e.m. Two-way ANOVA with Bonferroni correction was used for statistical analysis. ***p < 0.001.

Since RAS-dependent signaling is enhanced in NF1-DNFs, the activation status of RAS (RAS-GTP) and RAS-regulated pathways were examined. Following *KIR2DL5* knockdown (*KIR2DL5A/B*^KD^), there was a 3.5- (sh#1), 4.7- (sh#2), and 2.9- (sh#3) fold increase in RAS activation (RAS-GTP; Figure 4A). While others have shown that NF1-DNF growth depends on ERK1/2 pathway activation [30, 31], *KIR2DL5* reduction had no effect on ERK1/2^Thr202/Tyr204^phosphorylation (activation; Supplementary Figure 5A–5D), but rather led to increased AKT activation (AKT^Thr308^ and AKT^Ser473^ phosphorylation; sh#1, 31.5-fold and 2.8-fold; sh#2, 19.6-fold and 5.9-fold; sh#3, 15.6-fold and 7.3-fold, respectively; *n* = 3), as well as elevated mTOR activation (S6^Ser240/244^ phosphorylation; sh#1, 3.2-fold; sh#2, 4.9-fold; sh#3, 7.2-fold; *n* = 3) (Figure 4A). Similarly, expression of the mutant KIR2DL5 protein (*KIR2DL5 N173D*) resulted in 2.24-fold and 1.78-fold increases in AKT and S6 phosphorylation, respectively (Supplementary Figure 3).

To demonstrate that KIR2DL5 regulates cell proliferation in an AKT/mTOR-dependent fashion, we initially employed an AKT-specific inhibitor (MK2206). Following a 4h MK2206 treatment of control- and shRNA-infected Schwann cells, AKT^Ser473^ phosphorylation (sh#1, 0.9; sh#2 0.95; sh#3 0.97-fold), as well as the phosphorylation of two of its downstream effectors S6 (sh#1, 0.4; sh#2 0.6; sh#3 0.5-fold) and PRAS40 (sh#1, 0.7; sh#2, 1.3; sh#3, 1.2-fold), were reduced to control levels (Figure 4B). Additionally, AKT inhibition restored cell numbers (Figure 4C) and proliferation (Figure 4D) to normal levels. Next, to determine whether mTOR inhibition could similarly reverse the effects of KIR2DL5 KD, shRNA-infected Schwann cells were treated with rapamycin for 4h. Following rapamycin exposure, there was reduced AKT^Ser473^ phosphorylation (sh#1, 0.7; sh#2 1.1; sh#3 1.2-fold) and S6 phosphorylation (sh#1, 0.8; sh#2 0.8; sh#3 0.8-fold) (Supplementary Figure 5E), as well as restoration of cell numbers (Figure 4E) and proliferation (Figure 4F) to control levels. Collectively, these data establish that *KIR2DL5*-regulated Schwann cell growth activates RTK/AKT/mTOR signaling, raising the intriguing possibility that Sp-DNFs and NF1-DNFs converge on hyperactivation of the same mitogenic signaling pathway.

**Figure 5:**
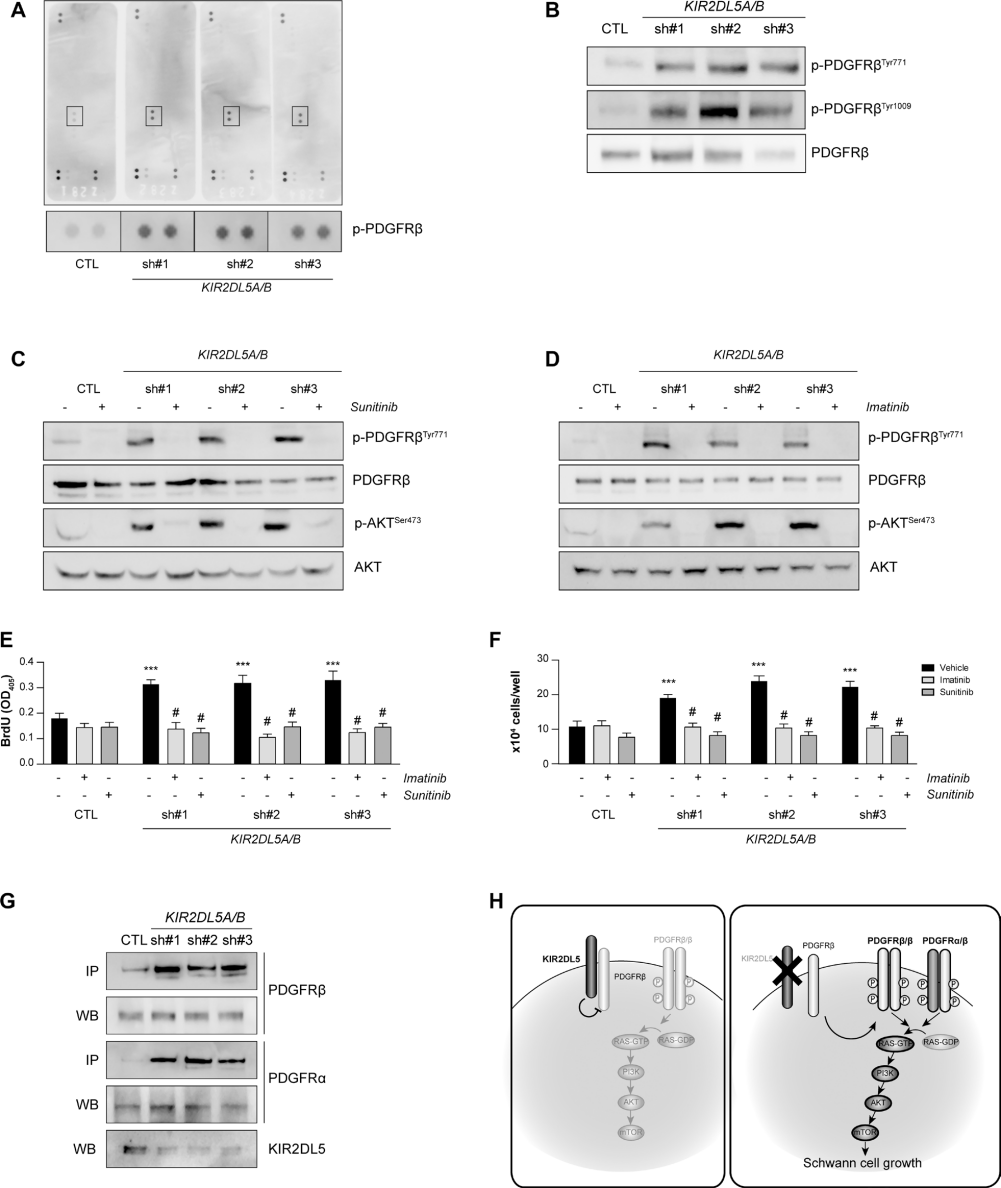
KIR2DL5A/B inhibits Schwann cell proliferation in a PDGFRβ/AKT-dependent manner (**A**) PDGFRβ is hyperphosphorylated following silencing of *KIR2DL5* in normal human Schwann cells as demonstrated with a human phospho-RTK array. (**B**) PDGFRβ^Tyr771^ and PDGFRβ^Tyr1009^ phosphorylation is increased following *KIR2DL5A/B* silencing. Inhibition of aberrant PDGFRβ and AKT phosphorylation following 16 h imatinib (**C**) or sunitinib (**D**) treatment restores cell numbers (**E**) and proliferation (**F**) to control levels. Data are represented as means ± s.e.m. All experiments were repeated at least three times. Two-way ANOVA with Bonferroni correction was used for statistical analysis. ***p < 0.001 compared to vehicle control, *p* < 0.001 compared to vehicle sh#1, sh#2, sh#3, respectively. (**G**) *KIR2DL5A/B* silencing leads to increased PDGFRβ/PDGFRα binding, as assessed by immunoprecipitation. (**H**) The proposed model of KIR2DL5 growth regulation envisions that KIR2DL5 normally binds and sequesters PDGFRβ, such that KIR2DL5 loss leads to increased PDGFRα/β binding and RAS/AKT-mediated hyperproliferation.

RAS/AKT signaling is typically initiated by receptor tyrosine kinase (RTK) receptor engagement in Schwann cells [32–36]. In order to identify this hyperactivated RTK, a commercially-available human phospho-RTK array was employed, which recognizes 49 distinct RTKs (Figure 5A). Whereas the activity of most RTKs was not significantly altered (*n* = 48 RTK moieties), platelet-derived growth factor receptor beta (PDGFRβ) phosphorylation was elevated (sh#1, 3.5-; sh#2 3.9-; sh#3, 3.5-fold) in *KIR2DL5A/B*^KD^ Schwann cells relative to controls. Independent validation of two of these activation-specific phosphorylation sites (PDGFRβ^Tyr771^: sh#1, 2-; sh#2, 4.5-; sh#3, 7.7-fold; and PDGFRβ^Tyr1009^: sh#1, 2-; sh#2, 5.4-; sh#3, 7.3-fold) confirmed receptor activation in *KIR2DL5A/B*^KD^ Schwann cells (Figure 5B). Consistent with a dominant mechanism of action of the *KIR2DL5 N173D* mutation, there was a 1.84-fold activation of PDGFRβ in *KIR2DL5 N173D*-expressing Schwann cells (Supplementary Figure 3). To determine the necessity of PDGFRβ activation for *KIR2DL5A/B*^KD^-mediated Schwann cell growth, we employed two different tyrosine kinase inhibitors that target PDGFRβ activation (imatinib [37–42] and sunitinib [43]), both of which are currently in clinical trials for the treatment of NF1-plexiform neurofibromas (Figure 5C–5F). Incubation of *KIR2DL5A/B*^KD^Schwann cells for 16h with either inhibitor reduced PDGFRβ and AKT hyperactivation (Figure 5C–5D), as well as restored total cell numbers (Figure 5E) and cell proliferation (Figure 5F) to control levels.

While increased PDGFRβ activation was responsible for AKT-mediated hyperproliferation following KIR2DL5 loss, the mechanism by which KIR2DL5 regulates PDGFRβ is not clear. We hypothesize that KIR2DL5 binds PDGFRβ to restrict its activation. Consistent with this model, KIR2DL5 binds PDGFRβ in normal human Schwann cells by PDGFRβ antibody-mediated immunoprecipitation. This binding is ameliorated in *KIR2DL5A/B*^KD^ Schwann cells, resulting in a 7.8-fold increase in PDGFRβ binding to PDGFRα (sh#1, 5.2-; sh#2, 9.6-; sh#3, 8.7-fold increase; Figure 5G). As such, KIR2DL5 likely inhibits PDGFRβ through direct binding, which reduces PDGFR heterodimerization, PDGFRβ phosphorylation and downstream RAS/AKT-mediated Schwann cell growth (Figure 5H).

Taken together, we identified the first mutation associated with sp-DNFs. This *KIR2DL5 N173D* mutation is hypothesized to result in attenuated KIR2DL5 protein levels, and as such, KIR2DL5 functions as a negative regulator of Schwann cell proliferation. In this regard, KIR2DL5 reduction, resulting either from *KIR2DL5* knockdown or *KIR2DL5 N173D* mutation, increases Schwann cell growth in a PDGFR/AKT/mTOR-dependent mechanism. Moreover, these studies provide the first demonstration that KIR2DL5 can function outside the immune system. Finally, while the etiologic mechanisms underlying sp-DNF and NF1-DNF growth control are distinct, they share a common signaling pathway amenable to therapeutic targeting.

## MATERIALS AND METHODS

### Tissue collection and processing

De-identified 10μm-thick sections of formalin-fixed paraffin-embedded DNF tissue from 7 male patients with a confirmed NF1 diagnosis and from 10 male patients with sp-DNFs were collected from Washington University Surgical Pathology Department. Total genomic DNA was extracted following review by an experienced neuropathologist (S.D.) using a QIAamp DNA FFPE Tissue Kit (QIAGEN) following manufacturer's instructions. Genomic DNA resuspended in elution buffer was sent for exome sequencing. Since this study was performed using de-identified dermal neurofibroma specimens and the patients could not be re-consented, we were unable to obtain blood for constitutional DNA analysis.

### Whole-exome sequencing and filtering

Whole human exome sequencing was performed from total genomic DNA of 17 tumors by Otogenetics Co. Briefly, library preparation was performed using an Illumina platform. Pair-ended sequencing (100 bp) with a minimum average coverage of 30x or ~2.7–3Gb was performed on a HiSeq2500 platform (human exome V5). Sequence reads were mapped to human reference genome hg18. Variants were annotated in Annovar and were filtered in Microsoft Excel to include only the following criteria: (1) Exonic variations (2) Non-synonymous variations, stop loss/ stop gain, frameshift insertions and frameshift deletions (3) 1000 genome, ExAc and ESP6500 frequencies ≤ 0.01 (4) Deleterious variants according to SIFT, Polyphen and CADD predictions (5) Non-common polymorphism in Cosmic and ClinVar (Supplementary Table 1). Genes mutated more than twice per tumor or resulting in conservative amino acid changes were eliminated from further analysis. cBioPortal for Cancer Genomics (www.cbioportal.org) was used to search recurrent identified mutations in other cancers. The complete exome sequencing data is in the process of being uploaded to dbGaP. The sequence reads were as follows:

**Table:** 

Sample	Size	Reads
Sp1	1,016,288,144	9,587,624
Sp2	2,980,285,61	28,115,902
Sp3	4,023,566,444	37,958,174
Sp4	2,015,092,012	19,010,302
Sp5	2,763,643,024	26,072,104
Sp6	2,264,326,420	21,361,570
Sp7	3,049,257,056	28,766,576
Sp8	2,111,053,38	19,915,598
Sp9	850,889,772	8,027,262
Sp10	4,016,548,608	37,891,968
NF1-1	1,752,023,756	16,528,526
NF1-2	3,296,901,464	31,102,844
NF1-3	2,697,579,372	25,448,862
NF1-4	2,130,578,164	20,099,794
NF1-5	1,632,340,852	15,399,442
NF1-6	1,685,491,160	15,900,860
NF1-7	2,751,629,620	25,958,770

### Immunohistochemistry and immunofluorescence

Paraffin-embedded tonsil, 4 normal peripheral nerves (sural nerve), 23 NF1-DNFs (13 males, 10 females) and 55 sp-DNFs (31 males, 24 females) were included in the immunohistochemical analyses. All DNFs were incubated with neurofibromin (Santa Cruz sc-67) antibodies, while and all DNFs, tonsil and normal sural nerve sections were incubated with KIR2DL5 (Abcam; ab175895) antibodies. Biotinylated secondary rabbit antibodies (Vector Laboratories) were used in combination with Vectastain Elite ABC development and hematoxylin counterstaining. Normal human tonsil was used as the reference control tissue for the KIR2DL5 antibody. Images were acquired on a Nikon Eclipse E600 microscope conjugated with a Nikon Plan Fluor 10x/0.30 DIC L objective and a Leica EC3 camera. Normal human sural nerve, sp-DNF sections, as well as primary cell cultures of normal human Schwann cells and HEK293T cells were analyzed by immunofluorescence using S100β (Millipore CB1040), c-Kit (Millipore; MAB1164), tryptase (Abcam; ab2378), vimentin (DSHB; AMF-17b) and KIR2DL5 (Abcam; ab129751) antibodies conjugated to appropriate Alexa Fluor secondary antibodies. Images were acquired on a Nikon Eclipse TE300 fluorescence microscope conjugated with a Nikon Plan Fluor 20x/0.45 ELWD objective and a Leica DFC3000G camera.

### Normal human Schwann cell culture, lentiviral infection and pharmacological inhibitor treatments

Normal human Schwann cells (ScienCell Research Laboratories) were cultured on poly-L-lysine-coated plates in Schwann cell medium (ScienCell #1701) supplemented with Schwann cell growth factors, Pen/Strep and fetal bovine serum (FBS), unless otherwise stated and were maintained as per the manufacturer's instructions. For serum-starvation experiments (Supplementary Figure 5), Schwann cells were plated on poly-L-lysine-coated plates in the presence of serum (FBS) for 16h, followed by a 36h period of serum deprivation during which cells were cultured only in Schwann cell medium supplemented with Schwann cell growth factors and Pen/Strep. 10% FBS was added to the cells for 2, 5, 15, 30 or 60 minutes at which time points cells were collected for western blotting analysis.

Three sets of lentiviral infection experiments were performed. Schwann cells were either infected once with lentivirus particles encoding (1) either a *KIR2DL5*^WT^ control or *KIR2DL5N173D* to establish the effect of the identified mutation on normal Schwann cells (Figure 2), (2) either a GFP-control or sh*KIR2DL5* RNA to knock down KIR2DL5 expression (Figures 3, 4, 5), or were infected twice with lentiviral preparations encoding (3) both a GFP control and *KIR2DL5*^CTL^ control or both sh*KRI2DL5* RNA and *KIR2DL5*^CTL^ to confirm the specificity of the shKIR2DL5 RNA constructs (Supplementary Figure 4). For all lentiviral infections, HEK-293T cells were initially cultured in DMEM (Gibco) supplemented with 10% heat-inactivated FBS and 1% Pen/Strep solution (Gibco). Three micrograms of total DNA was transfected per well of a 6-well plate (1.5 μg *KIR2DL5WT*, *KIR2DL5CTL KIR2DL5N173D, shKIR2DL5* or pLKO-GFP control; 1.5 μg lentiviral packaging constructs) using Fugene HD reagent (Promega) following manufacturer's instructions. 24h post transfection the media was replaced with fresh Schwann cell media supplemented with10% FBS, Schwann cell growth factors and antibiotics. Viral preparations were collected at 48 and 72 h post-transfection, were filtered, supplemented with polybrene and used for direct infections of Schwann cells. The sequences of the siRNA constructs were:

sh*KIR2DL5* #1: TCTCTCCATGACTCACCCTAT; sh*KIR2DL5* #2: CAGGAGCTCATTTGACATGTA; sh*KIR2DL5* #3: GAAACTCTTCAAGTAGTTCAT; KIR2DL5^WT^ cDNA cloned in a lentiviral vector was purchased from Origene and was either directly transfected into HEK293T cells, or was transfected following site-directed mutagenesis with QuikChange Site-Directed mutagenesis kit (Promega) following manufacturer's instructions in order to generate KIR2DL5^CTL^ (harboring six distinct base pair mutations within the short hairpin recognition sequences but resulting in identical amino acid sequence) or KIR2DL5^N173D^. All resulting constructs were subcloned and sequenced by Genewiz. The following primers were used to generate the expression constructs: *KIR2DL5*^CTL1^ (Forward: 5′-GA GCTTGGTTCAGTGGGTGAAGAAGAGCTGCTCGA GGAATTTCCTGTGACAGAAACAAGCAGTGG-3′, Reverse: 5′-CCACTGCTTGTTTCTGTCACAGGAAA TTCCTCGAGCAGCTCTTCTTCACCCACTGAACCA AGCTC-3′); *KIR2DL5*^CTL2^ (Forward: 5′- CCTACACAT GCTTCGGCTCGTTACATGATTCACCGTACGAGTG GTCAGACCCGAGTG-3′, Reverse: 5-CACTCGG GTCTGACCACTCGTACGGTGAATCATGTAACGAGC CGAAGCATGTGTAGG-3′); *KIR2DL5*^N173D^ (Forward: 5′-CCTGGAATGTTCCATCGACGCTGGGCACTGC-3′; Reverse: 5′-GCAGTGCCCAGCGTCGATGGAACAT TCCAGG-3′).

GFP control- or siRNA-infected Schwann cells underwent treatment with the following reagents: MK2206 (50nM in DMSO, 4 h; Selleckchem), rapamycin (10μM in DMSO, 4 h; Selleckchem), imatinib (10 μM in water, 16h; Sigma), sunitinib (10 μM in DMSO, 16 h; Sigma), or PD0325901 (10 nM in DMSO, 4h; Selleckchem).

### Western blotting, immunoprecipitation, RTK arrays and RAS/RAS pathway activity assays

Western blotting was performed on Schwann cells lysed in buffer containing 1% NP-40 (nonyl phenoxypolyethoxylethanol), supplemented with protease and phosphatase inhibitors using KIR2DL5 (Abcam), α–tubulin (Sigma), RAS (Millipore), phospho-AKT^Thr308^, phospho-AKT^Ser473^, AKT, phospho-^S6Ser240/244^, S6, phospho-Erk1/2^Thr202/204^, Erk1/2, phosphoPRAS40^Thr246^, PRAS40, phospho-PDGFRβ^Tyr771^, phospho-PDGFRβ^Tyr1009^, PDGFRβ, PDGFRα (Cell Signaling) primary antibodies, as well as HRP-conjugated secondary antibodies (Cell Signaling) and ECL (Fisher) chemiluminescence. Western signal band intensity was quantified using ImageJ Software (National Institutes of Health, USA, http://imagej.nih.gov/ij Java 1.7.0_67). *KIR2DL5A/BKD* cell KIR2DL5 expression levels (Figures 2A, 3D) were calculated by normalizing the relative KIR2DL5/α-tubulin band intensity ratio to that of GFP-control-infected Schwann cells. For the immunoprecipitation studies, 100 μg total Schwann cell protein lysate was pre-cleared for 1h in agarose protein G beads (Cell Signaling), immunoprecipitated with PDGFRβ overnight and collected with agarose protein G beads for 2 hours. For the human phospho-RTK array (human phospho-receptor tyrosine kinase array kit; R&D Systems) Schwann cells were lysed in the provided buffer and 80μg total protein was assayed using manufacturer's instructions. In brief, the assay can detect phosphorylation of the following RTKs: ALK/CD246, EphB4, MuSK, Axl, EphB6, PDGFRα, DDR1, ErbB2, PDGFRβ, DDR2, ErbB3, c-Ret, Dtk, ErbB4, ROR1, EGFR, FGFR1, ROR2, EphA1, FGFR2α, Ryk, EphA2, FGFR3, SCFR/c-kit, EphA3, FGFR4, Tie-1, EphA4, Flt-3/Flk-2, Tie-2, EphA5, HGFR/c-MET, TrkA, EphA6, IGF1R, TrkB, EphA7, Insulin R/CD220, TrkC, EphA10, M-CSFR, VEGFR1/Flt-1, EphB1, Mer, VEGFR2/KDR, EphB2, MSPR/Ron, VEGFR3/Flt-4, and EphB3. Since only PDGFRβ was hyperphosphorylated, the activation was independently validated with two separate phospho-PDGFRβ antibodies: phospho-PDGFRβ^Tyr771^, phospho-PDGFRβ^Tyr1009^ (Cell Signaling). RAS activity assays were performed following Raf1-RBD immunoprecipitation using manufacturer's instructions (RAS activation kit; Millipore). All experiments were repeated at least three times using independently derived cell lysates.

### Proliferation and apoptosis assays

Schwann cell proliferation and TUNEL staining were assessed using the BrdU Cell Proliferation Colorimetric ELISA kit (Roche) or the TUNEL staining kit (Roche) following manufacturer's instructions. For both assays, Schwann cells were seeded at 5000 cells/ well of a 96-well plate. For the proliferation assays, Schwann cells were serum-starved for 24 hours, if treated, incubated in vehicle or appropriate inhibitors for a total of 24 hours, labelled with BrdU for 18 h followed by 90min incubation in peroxidase-conjugated anti-BrdU antibody. The optical density of the cells was measured at 405 nm on a Bio-Rad spectrophotometer. For the TUNEL assay, cells were incubated in serum-containing media for 24 h before being fixed in 4% PFA, incubated in TUNEL substrate for 1 h and imaged using a fluorescent microscope.

### Statistical analyses

All statistical tests were performed using GraphPad Prism 5 software. Student's *t*-test, one-way or two-way analysis of variance (ANOVA) with Bonferroni pos*t*-test correction was employed for all experiments.

## SUPPLEMENTARY MATERIALS FIGURES AND TABLES




