# Supplementary material for: KIR2DL5 mutation and loss underlies sporadic dermal neurofibroma pathogenesis and growth
Source: Oncotarget. 2017 May 10;8(29):47574–85. doi: 10.18632/oncotarget.17736 (PMC5564588; doi:10.18632/oncotarget.17736)
Supplement: Supplementary file 1 [file oncotarget-08-47574-s001.pdf]

KIR2DL5 mutation and loss underlies sporadic dermal neurofibroma pathogenesis and growth

Supplementary Materials

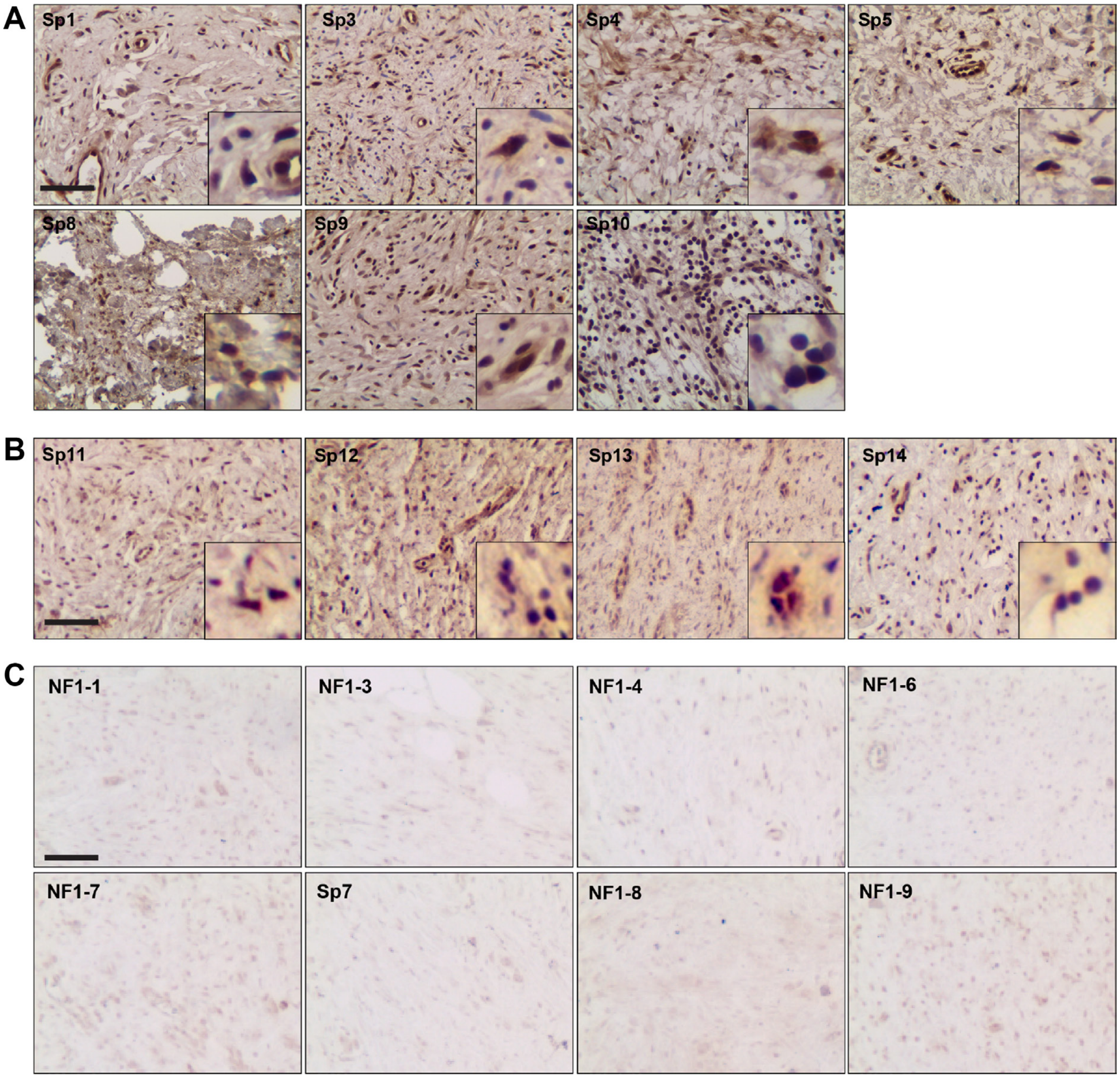

Supplementary Figure 1: Neurofibromin expression in sporadic and NF1-DNFs. (**A–C**) Immunohistochemical detection of neurofibromin expression in (C) sequenced sporadic, (B) independently-acquired, unrelated sp-DNFs (four representative samples shown), and (C) NF1-DNFs. Sp, sporadic DNF (*n* = 55 individual tumors); NF, NF1-associated DNF (*n* = 23 individual tumors; representative samples shown). One sporadic DNF lacked neurofibromin expression (Sp7).

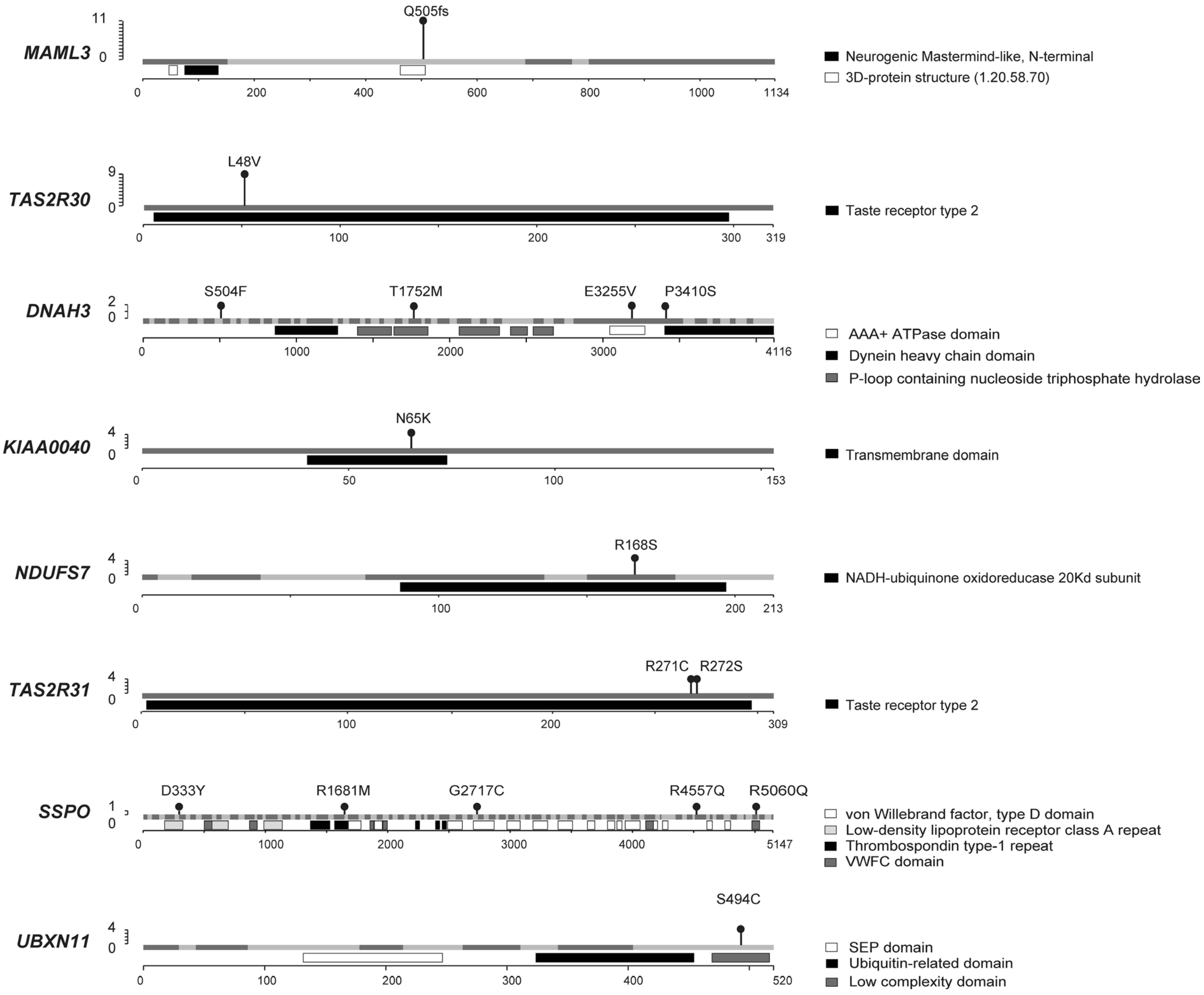

Supplementary Figure 2: Schematic representation of single nucleotide variations in genes commonly mutated in NF1-associated and sporadic DNFs. Lollipop height indicates the number of tumors in which each mutation was identified.

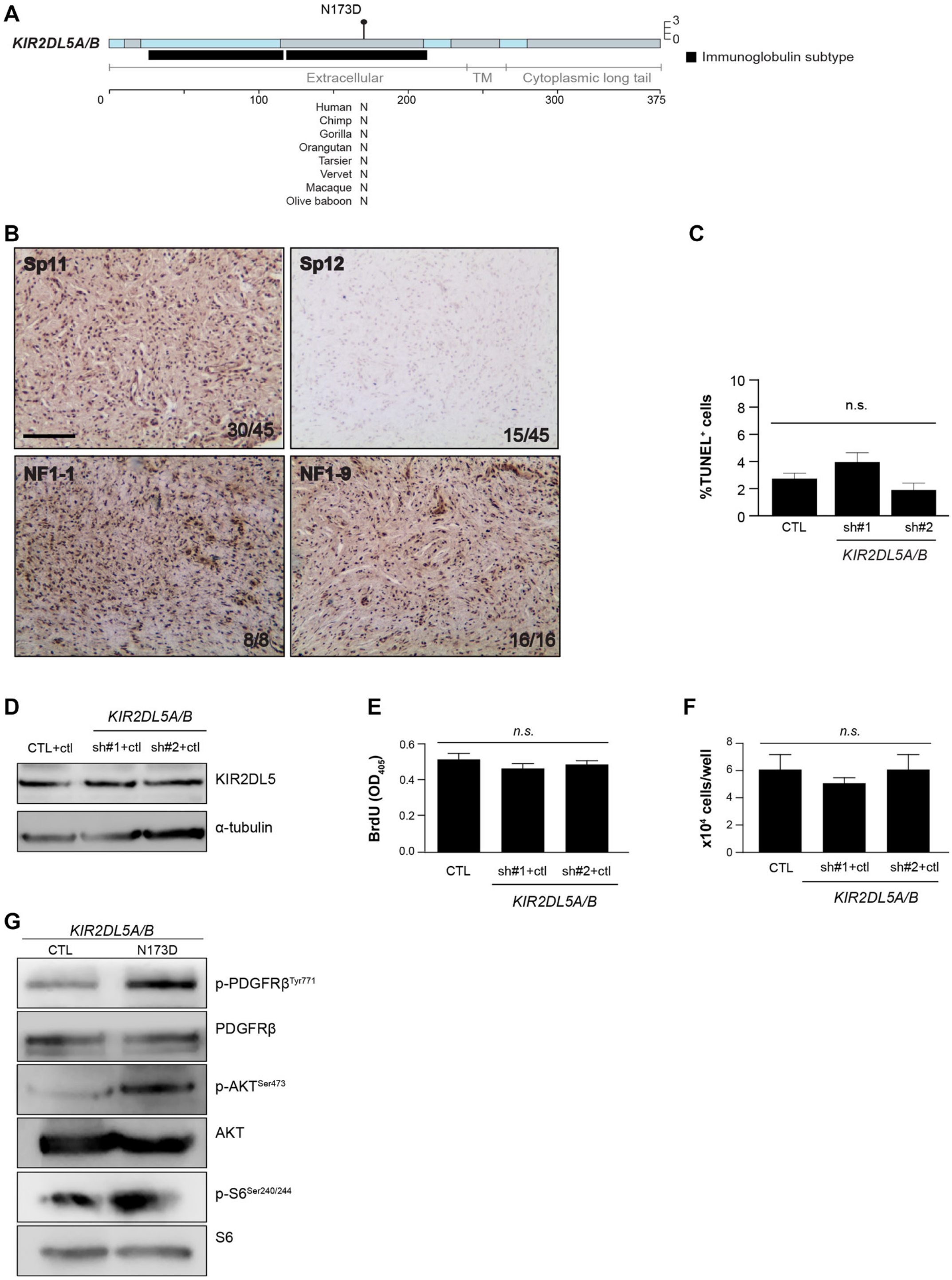

Supplementary Figure 3: A non-conservative missense mutation in the KIR2DL5 gene was identified in sp-DNFs. (**A**) The Asn173Asp (N173D) mutation lies within a highly-conserved immunoglobulin subtype domain shared across human and non-human primates. (**B**) KIR2DL5 expression in an independent series of 45 sp-DNFs and 24 NF1-DNFs (8 sequenced and 16 independent NF1-DNFs). One representative immunopositive (Sp11) and one immunonegative (Sp12) sp-DNF are shown, as well as representative immunopositive (NF1-1 and NF1-8) NF1-DNFs. (**C**) Apoptosis (%TUNEL$^+$ cells) is similar in human Schwann cells infected with GFP-control (CTL) or two independently-generated *KIR2DL5* shRNA constructs. (**D**–**F**) Wild-type *KIR2DL5* (*KIR2DL5*^{WT}) expression in human Schwann cells with *KIR2DL5* shRNA-mediated knockdown restores KIR2DL5 expression, proliferation and cell numbers relative to KIR2DL5 knockdown alone (Figure 3). (**G**) Expression of the mutant *KIR2DL5* gene (*KIR2DL5*^{N173D}) results in increased PDGFRβ, AKT and S6 activation. One-way ANOVA was used for statistical analysis. Data are represented as the means ± s.e.m. n.s., not significant. Scale bar, 25 µm.

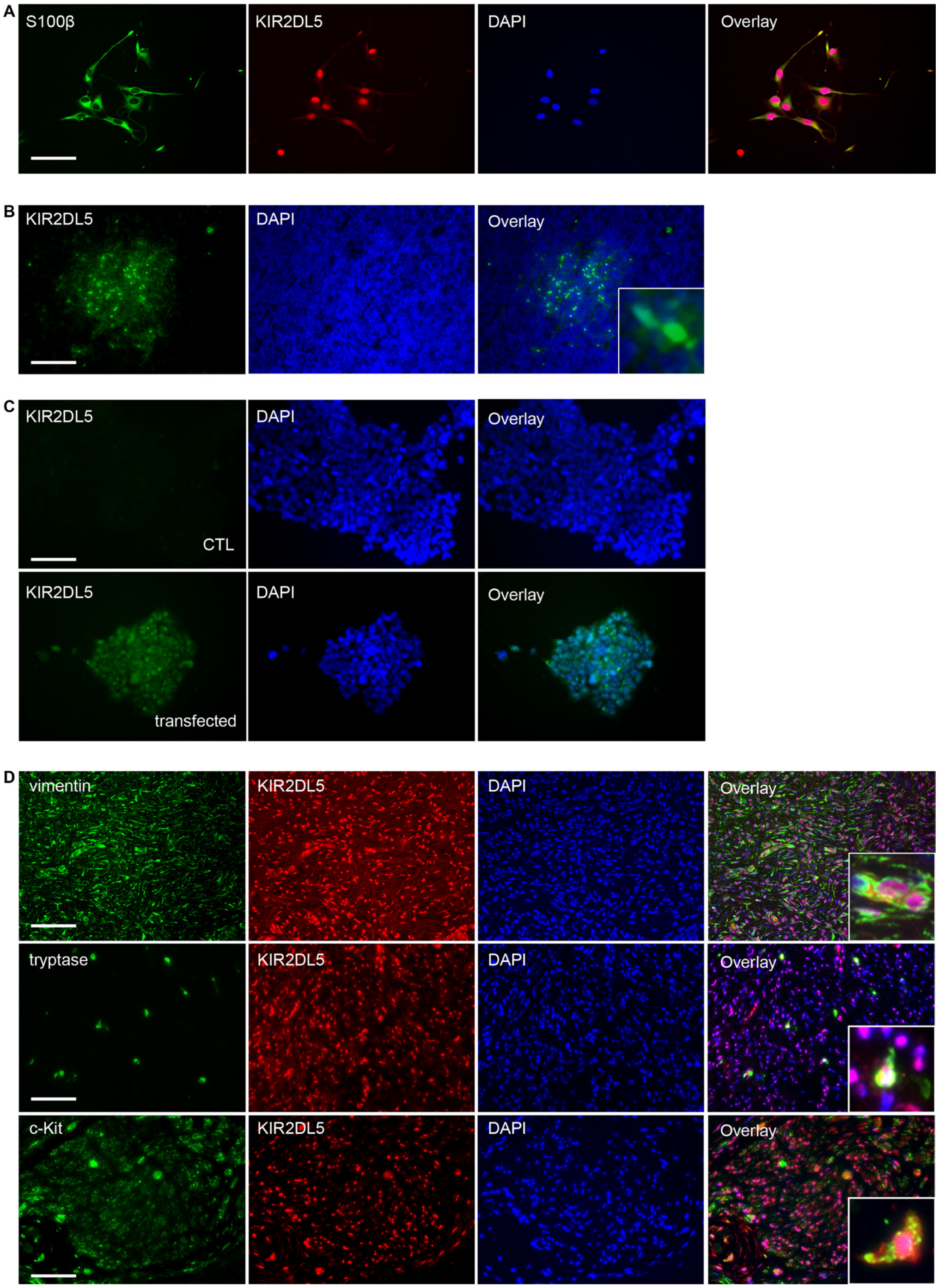

Supplementary Figure 4: KIR2DL5 expression in primary cells. (**A**) Co-labelling of KIR2DL5 and S100β in normal human Schwann cells reveals both nuclear and perinuclear KIR2DL5 subcellular localizations. (**B**) Labelling of KIR2DL5 in the manufacturer's (Abcam) recommended control tissue (tonsil) reveals KIR2DL5 expression in both the nucleus and perinuclear region. (**C**) HEK293T cells lack endogenous KIR2DL5 expression (CTL; top panel), but demonstrate nuclear and perinuclear KIR2DL5 expression following wild-type KIR2DL5 transduction (transfected; bottom panel). (**D**) Expression of KIR2DL5 in mast cell (tryptase^{+}, c-Kit^{+} cells) and fibroblasts (vimentin^{+} cells). Scale bar, 25 µm.

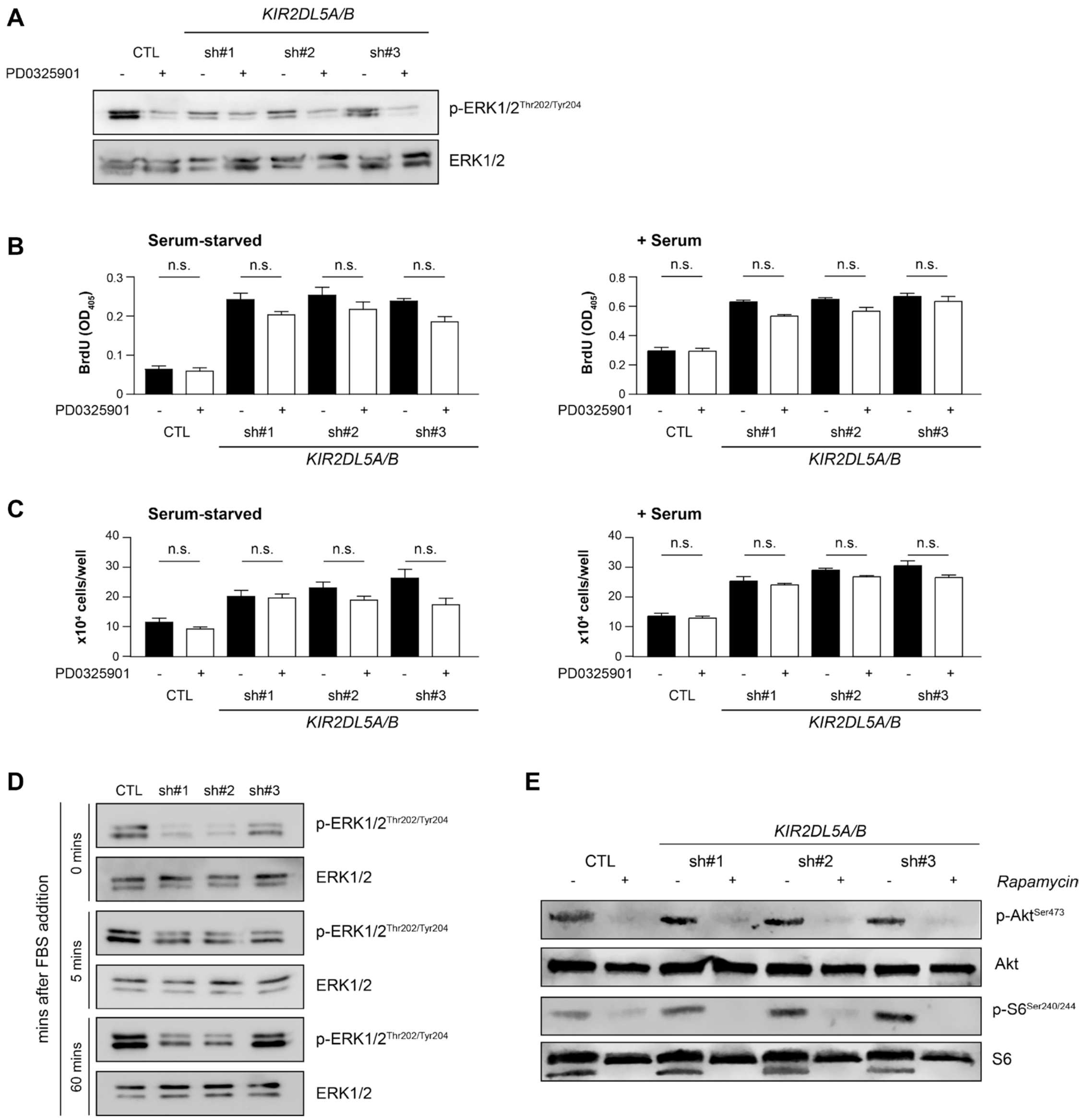

Supplementary Figure 5: KIR2DL5^{KD} Schwann cell growth is ERK-independent. (**A**) ERK1/2 activation (phosphorylation) is attenuated following PD0325901 treatment as detected by Western blot. (**B**, **C**) Schwann cell BrdU incorporation (B) and total Schwann cell numbers (C) are not altered following pharmacological MEK inhibition (PD03225901; 10 nM), either in the presence (right panels) or absence (left panels) of serum. (**D**) ERK1/2 activation is decreased in *KIR2DL5*^{KD} Schwann cells relative to controls, either in the presence or absence of serum. (**E**) AKT and S6 hyper-phosphorylation in *KIR2DL5*^{KD} Schwann cells is restored to control levels following a 4 h Rapamycin treatment. The arrow denotes the total S6 protein (predicted molecular size = 32kDa).

Supplementary Table 1: List of identified gene mutations in the 17 DNFs studied following initial filtering. See_Supplementary_Table_1
